# Announcements

**Published:** 2015-04-24

## World Malaria Day — April 25, 2015

World Malaria Day is commemorated on April 25, the date in 2000 when 44 African leaders met in Abuja, Nigeria, and committed their countries to reducing malaria-related deaths. During 2000–2013, the scale-up of effective malaria prevention and control interventions saved an estimated 4.2 million lives, with 92% of those being children aged <5 years, and decreased malaria mortality by 30% globally and 34% in sub-Saharan Africa (1). In spite of these accomplishments, an estimated 198 million cases of malaria occurred globally in 2013, resulting in an estimated 584,000 deaths (1).

In recent years, there have been increases in resistance to mosquito insecticides and treatment drugs and changes in malaria epidemiology as a result of scaled-up interventions. Thus, new approaches are needed to sustain progress in malaria control and to move beyond control to malaria elimination. The theme of World Malaria Day 2015 is Invest in the Future: Defeat Malaria.

CDC supports global malaria control efforts through the President’s Malaria Initiative, a U.S. government interagency initiative to reduce malaria incidence and mortality in 19 countries in sub-Saharan Africa and in the Greater Mekong Subregion in Asia. This effort has helped deliver millions of insecticide-treated mosquito nets, antimalarial drugs, and rapid diagnostic test kits to ensure that persons at risk for malaria will have access to lifesaving prevention and treatment. CDC also conducts multidisciplinary strategic and applied research globally to increase knowledge about malaria and develop safe, effective interventions that can lead to the elimination and eventual eradication of malaria worldwide.

Through a grant to the CDC Foundation from the Bill and Melinda Gates Foundation, CDC is also leading a consortium of malaria partners, known as the Haiti Malaria Elimination Consortium, aiming to eliminate indigenous cases of malaria on the island of Hispaniola by 2020. Additional information regarding CD’s malaria activities is available at http://www.cdc.gov/malaria.
