# Errata

**Published:** 2015-05-01

## Vol. 64, No. 15

In the announcement “World Malaria Day — April 25, 2015” on page 425, errors occurred in the second sentence. The sentence should read as follows: “During 2000–2013, the scale-up of effective malaria prevention and control interventions saved an estimated 4.2 million lives, with 92% of those being children aged <5 years, and decreased malaria mortality by 47% globally and 54% in the African Region (1).”

